# Correlation between desynchrony of hippocampal neural activity and hyperlocomotion in the model mice of schizophrenia and therapeutic effects of aripiprazole

**DOI:** 10.1111/cns.14739

**Published:** 2024-05-03

**Authors:** Xueru Wang, Zijie Li, Shihui Kuai, Xuejiao Wang, Jingyu Chen, Yanping Yang, Ling Qin

**Affiliations:** ^1^ Department of Physiology China Medical University Shenyang Liaoning China; ^2^ Department of Anesthesiology Shengjing Hospital of China Medical University Shenyang Liaoning China

**Keywords:** aripiprazole, hippocampus, hyperlocomotion, neural activity, schizophrenia

## Abstract

**Aims:**

The hippocampus has been reported to be morphologically and neurochemically altered in schizophrenia (SZ). Hyperlocomotion is a characteristic SZ‐associated behavioral phenotype, which is associated with dysregulated dopamine system function induced by hippocampal hyperactivity. However, the neural mechanism of hippocampus underlying hyperlocomotion remains largely unclear.

**Methods:**

Mouse pups were injected with *N*‐methyl‐d‐aspartate receptor antagonist (MK‐801) or vehicle twice daily on postnatal days (PND) 7–11. In the adulthood phase, one cohort of mice underwent electrode implantation in field CA1 of the hippocampus for the recording local field potentials and spike activity. A separate cohort of mice underwent surgery to allow for calcium imaging of the hippocampus while monitoring the locomotion. Lastly, the effects of atypical antipsychotic (aripiprazole, ARI) were evaluated on hippocampal neural activity.

**Results:**

We found that the hippocampal theta oscillations were enhanced in MK‐801‐treated mice, but the correlation coefficient between the hippocampal spiking activity and theta oscillation was reduced. Consistently, although the rate and amplitude of calcium transients of hippocampal neurons were increased, their synchrony and correlation to locomotion speed were disrupted. ARI ameliorated perturbations produced by the postnatal MK‐801 treatment.

**Conclusions:**

These results suggest that the disruption of neural coordination may underly the neuropathological mechanism for hyperlocomotion of SZ.

## Graphical abstract

Hyperlocomotion is a characteristic schizophrenia‐associated behavioral phenotype, which is linked to deregulated dopaminergic efflux induced by hippocampal hyperactivity. We reported how the disruption of neural coordination in dCA1 is involved in hyperlocomotion of postnatal MK‐801‐treated mice, which can be ameliorated by aripiprazole.

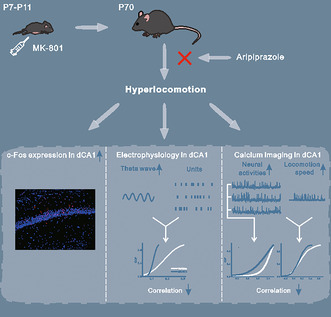

## 1 INTRODUCTION

Schizophrenia (SZ) is a one of the most common neuropsychiatric disorders, which affects approximately 1% of the world population.^1^, ^2^, ^3^ It is manifested by the presence of three core symptoms: positive symptom, negative symptom and cognitive deficits.^2^, ^4^ Hyperlocomotion is a characteristic behavioral phenotype associated with SZ, linked to dysregulated dopamine system function in subcortical regions such as the nucleus accumbens (NAc).^5^, ^6^ Hyperactivity of the hippocampus is believed to enhance dopamine efflux in the NAc,^7^, ^8^ and significant increases in glutamate levels and hyperactivity have been reported in the hippocampus of SZ.^9^, ^10^, ^11^, ^12^ However, the precise hippocampal neural mechanism underlying hyperlocomotion remains unclear.

To address this issue, it is necessary to conduct neurophysiological experiments on animal model of SZ. Because *N*‐methyl‐d‐aspartate receptors (NMDAR) are involved in the normal development of neural circuits, rodents administered NMDAR antagonists (MK‐801) during early developmental periods are well utilized as animal models for SZ.^13^, ^14^ This type of treatment impairs motor function, similar to it observed in SZ patients.^13^, ^15^ The firing rates of hippocampal CA1 neurons exhibited a significant linear correlation with the locomotion speed of rats.^16^ And a series of disruptions have been reported in the hippocampal CA1 region of MK‐801 model mice, including impaired synaptic plasticity,^17^ increased gamma oscillations and disrupted theta/gamma coupling.^18^

Here, we used in vivo electrophysiological recording and calcium imaging on the MK‐801‐induced model mice of SZ to evaluate the correlation between abnormalities of hippocampal CA1 neural activity and hyperlocomotion. In the electrophysiological recording experiment, we recorded local field potentials (LFPs) and single‐neuron spikes, which is an effective method for elucidating the ability of neural oscillations and coordinated assemblies of neurons. Calcium imaging with genetically encoded calcium indicators can simultaneously monitor individual neural activities, is optimal for investigating the neural activities during locomotion.

Furthermore, objectively evaluating therapeutic effects of antipsychotic drugs is also an important issue for SZ research. Aripiprazole (ARI) is an atypical antipsychotic drug with a unique mechanism of action comprising partial dopamine type2 (D2) and serotonin 5‐HT1A receptors agonism and antagonism at serotonin 5‐HT2A receptors,^19^ which is effective for improving symptoms associated with SZ.^20^ We do not yet know how this drug affects the activity of individual hippocampal neurons and the correlations between them. We, therefore, examined this by employing electrophysiological recording and calcium imaging on the hippocampus of model mice. We found that hippocampal neurons in the MK‐801‐induced SZ mice showed hyperactivity and dysrhythmia, which may correlate with hyperlocomotion; and these abnormalities were ameliorated by ARI. Our study provided a system‐level strategy to investigate the neuropathological mechanism of SZ and evaluate the therapeutic effects of ARI.

## 2 MATERIALS AND METHODS

### 2.1 Animals

C57BL/6 mice (Vital River laboratory, Beijing, China) were housed in group of 2–3 per cage with free access to food and water, and maintained on a 12:12 light/dark cycle. The animals were maintained and treated in compliance with the policies and procedures detailed in the “Guide for the Care and Use of Laboratory Animals” of the National Institutes of Health. All experiment procedures were approved by the Animal Ethics Committee (CEUA) of China Medical University. In total, 36 mice were used in the open‐field test (OFT), 46 mice in electrophysiological recordings, and 16 mice in calcium imaging. Mice in all experiments included both male and female.

### 2.2 Experimental procedures

On postnatal day (PND) 7–11, C57BL/6 mouse pups were randomly intraperitoneally (i.p.) injected with 0.5 mg/kg MK‐801 (Sigma‐Aldrich, St. Louis, MO, USA) freshly dissolved in 0.9% saline, or an equal volume of vehicle (saline) twice daily. After the injection, pups were returned to their cages and remained with their mother until the age of weaning (PND 21). On PND‐70, MK‐801‐treated and vehicle mice were subjected to OFT, electrophysiological recording and in vivo calcium imaging.

As referenced in previous study,^21^ we selected a dose of 1 mg/kg ARI (MedChemExpress, Monmouth Junction, NJ, USA) for administration (i.p.) to attenuate MK‐801‐induced hyperlocomotion. In the OFT, ARI was injected 30 min before the start of the test. For electrophysiological and calcium imaging experiments, ARI was injected after baseline recordings. After 3 days for washing out, drug injections were administered again, followed by repeated electrophysiological or imaging experiments. Each mouse received 2 rounds of ARI injection.

### 2.3 Open‐field test

The OFT was conducted in a 55 × 40 × 30 cm (length × width × height) arena constructed of non‐transparent black boards for well position tracking. Before testing, each mouse was brought into the procedure room singly and underwent 5 min of habituation in their home cage. Upon completion of the habituation period, the mouse was placed in the center of the open field arena. Results include total distance traveled and duration in center (located in the center of arena with 25% of total area). The total duration of OFT, excluding habituation, was 30 min.

### 2.4 Electrophysiological recordings

After recovery from the surgery, electrophysiological recordings were conducted in an electrically shielded, sound‐proof box. The mice were accustomed to head fixation and familiarized with the environment for 2 h where they were affixed atop a free‐spinning treadmill through the custom headposts on the skull. The mouse could move its body while the head was fixed. The recording experiments were performed on the 5th day.

In the experiments of multi‐channel probe recording, mice were briefly anesthetized by isoflurane, a small craniotomy was performed above the marked locations of hippocampus. The dura was removed and the craniotomy was protected with saline. After recovery from anesthesia, a multi‐channel silicon probe (A1x16‐3.8 mm‐50‐177, NeuroNexus, MI, USA) was mounted on a remotely controlled manipulator (MO‐10, Narishige, Tokyo, Japan) and gradually penetrated into the hippocampus to record LFP and extracellular spike activities. Raw signals were digitized with a multichannel extracellular amplifier (RA16PA; Tucker‐Davis Technologies, TDT; Alachua, FL, USA). And they were band‐pass filtered to extract LFP (1–300 Hz) and spike activity (300–5000 Hz), then imported into computer for further analysis. Single unit activity (SUA) was isolated using a wavelet‐based spike sorting package (OpenSorter software, TDT).

### 2.5 Calcium imaging

For awake imaging, we used a free‐spinning treadmill, which allowed imaging during running states. On experimental day: imaging started after 30 min of habituation in head fixation under the microscope for each mouse. The window was centered under the microscope. Images were acquired with a 4× objective (Laite Photoelectric Technology Co., LTD, Guangzhou, China). Image acquisition was carried out with a LED tuned to 485 nm to excite GCaMP6s. Single planes (1024 × 1024 pixels) were acquired at 10 Hz. For the repetitive imaging, the position of the field of view (FOV) was registered in the first imaging session with the help of vascular landmarks and cell bodies of CA1 pyramidal neurons. This allowed for subsequent retrieval of the FOV for each mouse. The treadmill running speed of mice was collected using a custom‐built software.

### 2.6 Analysis of electrophysiology data

LFP power and spike‐field coherence were analyzed using chronux toolbox implemented in custom‐written code (http://chronux.org/).^22^ For the analysis of spike activity, we computed the instantaneous firing rate (*spike count*/*bin duration*). The spike‐field coherence was determined in theta band (5 to 12 Hz) for each spike unit. To facilitate comparison across units, the same number of spikes was used for each unit (# of spikes from the unit with the fewest spikes) from randomly selected spikes. For each randomly selected spike during locomotion, 300 ms of raw LFP surrounding the event was averaged and the power spectral density was taken. This was then normalized to the average of each power spectrum used to generate the average signal, yielding a coherence value ranging from 0 to 100%. Theta band spike‐field coherence was defined by the maximum coherence value in the range of 5 to 12 Hz.

### 2.7 Analysis of calcium imaging data

Motion correction of GCaMP6s calcium imaging movies was performed offline using FIJI (National Institutes of Health, Bethesda, MD, USA) plugin “Image Stabilizer” (http://www.cs.cmu.edu/~kangli/code/Image_Stabilizer.html). After motion correction, signal extraction, correlation, and analysis for calcium signal was performed using Python (Python Software Foundation, New Hampshire, USA) in the Spyder (Pierre Raybaut, The Spyder Development Team) development environment. Calcium imaging data were analyzed with the Suite2p toolbox.^23^, ^24^

The number and height of calcium transient properties were calculated with the scipy function *find_peaks* on the raw calcium traces with the following parameters: height = 20, distance = 10, and prominence = 20. The decay was computed on the 10 best isolated transients of each neuron, using the OASIS toolbox (https://github.com/j‐friedrich/OASIS). We used the deconvolve function with the following parameters: penalty = 0 and optimize_g = 10. Traces with an estimated decay over 2.5 s were considered cases of failed extraction and removed from further analysis. Correlations were computed both as Pearson (numpy function corrcoeff) and Spearman (custom‐written function) coefficient on the *z*‐scored signal. To both sets of coefficients, the Fisher correction was applied.

### 2.8 Statistical analyses

Statistical analysis was performed using GraphPad Prism 9.0 (graphpad.com). Data were compared via *t*‐tests or two‐way ANOVA with Šídák's post‐hoc test for multiple comparisons. Values in the text are reported as mean ± standard difference (SD) unless reported otherwise. Results were considered statistically significant when the *p* value <0.05.

More detailed methods of experiment procedures, surgery and immunofluorescence are provided in Supplemental Materials.

## 3 RESULTS

### 3.1 Early postnatal MK‐801 treatment induces hyperlocomotion and increased expression of immediate early gene in hippocampal neurons

**FIGURE 1:**
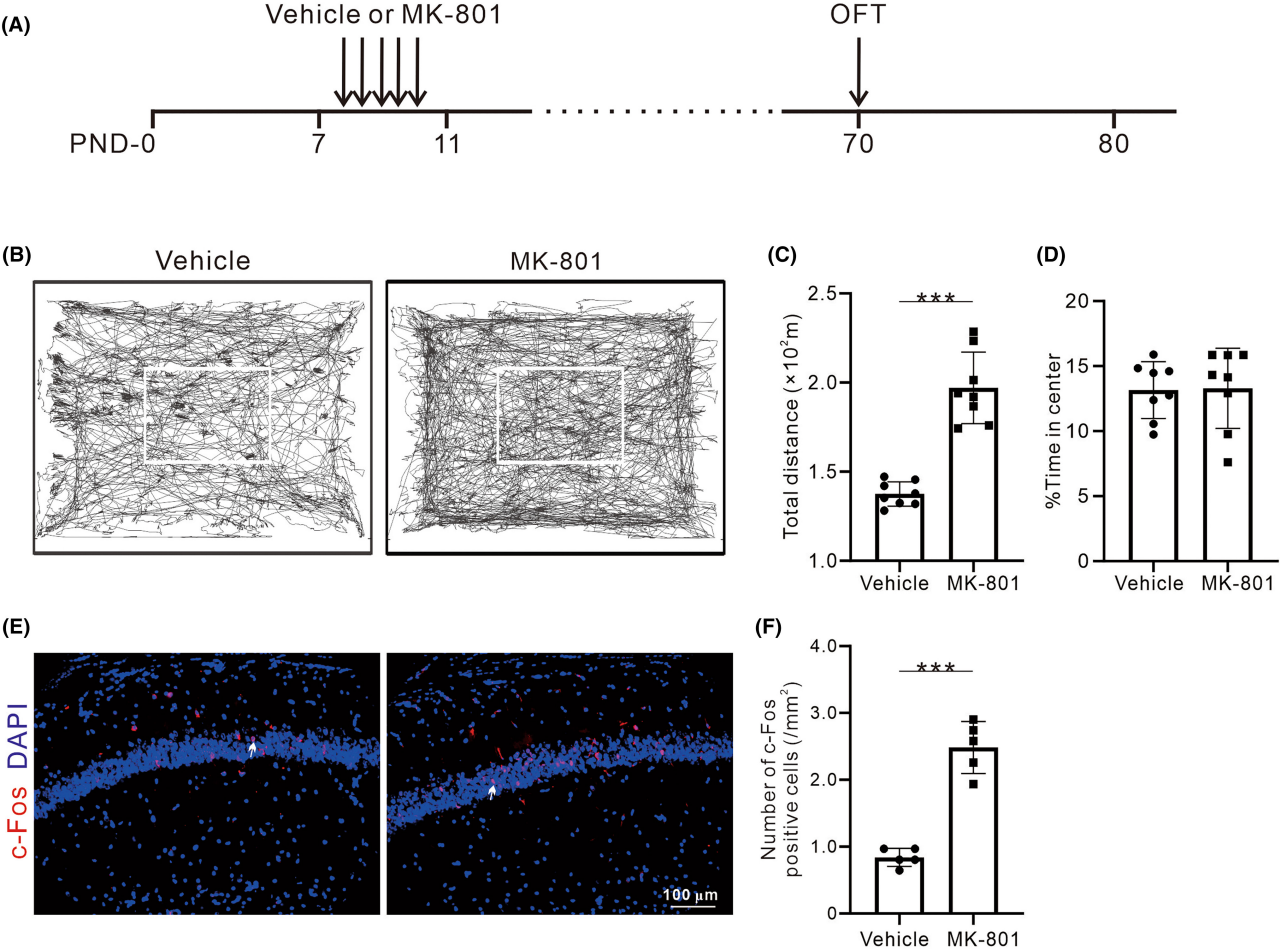
Effects of early postnatal MK‐801 treatment on locomotion and expression of c‐Fos in hippocampus. (A) The experimental timeline for vehicle or MK‐801 treatment and behavioral tests. (B) Movement trace diagrams of the vehicle and MK‐801‐treated mice in the OFT (the white box indicates the central area of the OFT). (C, D) Statistical charts of total traveled distance and the percent of time spent in the center in the OFT. Dots represent the values of individual mouse. Bars and ticks are means ± SD from eight mice (four male/four female) per group. (E) Representative images of c‐Fos (red) and DAPI (blue) in the dCA1, white arrows on c‐Fos/DAPI panel. (F) Quantification of the number of c‐Fos positive cells in dCA1 between vehicle and MK‐801‐treated mice. Values are from five mice (two male/three female) per group. *p < 0.05, **p < 0.01, ***p < 0.001, Student's *t*‐test.

To establish the model of SZ, PND‐7 mouse pups were intraperitoneally injected with MK‐801 or vehicle twice daily for 5 days. At adulthood (PND‐70), the MK‐801‐treated and vehicle mice were subjected to OFT to estimate locomotor behavior in the model of SZ (Figure 1A). In the OFT, MK‐801‐treated mice showed traveled more in both the center and peripheral area when compared to controls (Figure 1B), resulted in a dramatical increase in the traveled distance (*t*_14_ = 7.94, *p* < 0.001, Figure 1C). However, the percentage of time spent in the center area was not significantly changed, despite an elevated trajectory density in the center area (*t*_14_ = 0.10, *p* = 0.92, Figure 1D). The results indicated that MK‐801‐treated mice exhibited hyperlocomotion but not anxiety. Early postnatal MK‐801 treatment caused hyperlocomotion in the adulthood phase. To analyze hippocampal neuronal activity during locomotion, we evaluated the expression of c‐Fos, which is one of the immediate early genes. The results (Figure 1E,F) showed that the number of c‐Fos‐positive cell in the dCA1 was significantly increased in MK‐801‐treated mice (*t*_8_ = 8.95, *p* = <0.001).

### 3.2 Early postnatal MK‐801 treatment disrupts coupling between hippocampal spiking activity and theta oscillations

**FIGURE 2:**
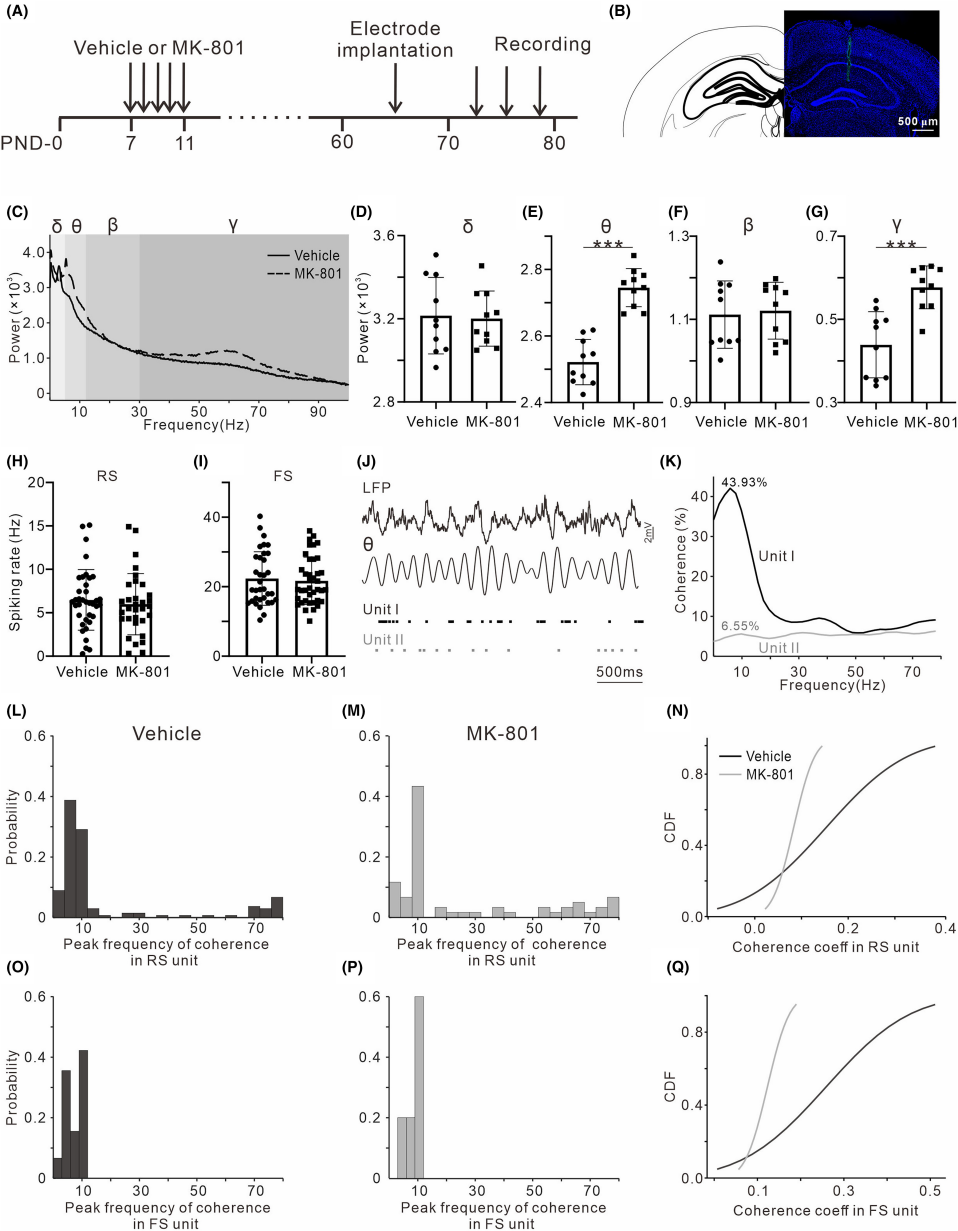
Effects of early postnatal MK‐801 treatment on hippocampal spiking activities and LFP oscillations. (A) The timeline of the experimental procedures for in vivo electrophysiological recording. (B) Histological sections showing the location of the multi‐channel probe. (C) The example power spectra of LFP recorded from one vehicle (black line) and MK‐801‐treated mouse (dashed line). Gray shadings indicate the frequency bands of delta, theta, beta and gamma. (D–G) Statistical charts of the power of delta, theta, beta and gamma bands (*n* = 10; five male/five female). *p < 0.05, **p < 0.01, ***p < 0.001, Student's *t*‐test. (H, I) Statistical charts of spiking rate for RS unit (*n* = 37/32 from 10 vehicle/MK‐801‐treated mice; five male/five female) and FS unit (*n* = 34/39 from 10 vehicle/MK‐801‐treated mice; five male/five female). (J) Example LFP and simultaneously recorded two spiking activities. Black dots show one spiking activity phase‐locking with respect to hippocampal theta oscillations, whereas the other represented in gray dots does not. (K) Line plot showing spike‐LFP coherence for two examples from (I). (L, M) Probability distribution histogram of the peak frequency of spike‐LFP coherence in RS unit. (N) Line plot displaying cumulative distribution of coherence coefficients in RS unit. (O, P) Probability distribution histogram of the peak frequency of spike‐LFP coherence in FS unit. (Q) Line plot displaying cumulative distribution of coherence coefficients in FS unit.

Using electrophysiological recording from the hippocampus of PND‐70 mice, we investigated how the MK‐801 treatment affects the correlation between the spiking activities of neurons and the oscillations of LFP (Figure 2A,B). First, Figure 2C shows the example power spectra of LFP recorded from hippocampus, and MK‐801 treatment significantly increased the powers of LFP in the theta (5–12 Hz) (*t*_18_ = 7.94, *p* < 0.001, Figure 2E) and gamma band (30–80 Hz) (*t*_18_ = 4.61, *p* < 0.001, Figure 2G), while did not affect those in the delta (1–4 Hz) (*t*_18_ = 0.19, *p* = 0.85, Figure 2D) and beta band (13–30 Hz) (*t*_18_ = 0.28, *p =* 0.78, Figure 2F).

Second, we extracted two classes of unit (RS and FS) based on their waveform shape. FSs were distinguished from RSs by their comparatively rapid time course, about half that of RSs. RSs discharged spontaneously at rates of less than 1–15/s, whereas FSs displayed rates of 15–50/s. The amplitudes of FSs, which were generally smaller than those of RSs, often decreased during high‐frequency discharges. No significant difference of spiking rate in the unit of RS (*t*_67_ = 0.59, *p* = 0.56, Figure 2H) and FS (*t*_71_ = 0.37, *p* = 0.71, Figure 2I) was found between the MK‐801‐treated and vehicle mice.

Third, we examined the coupling between spiking activities and theta oscillations. Figure 2J presents two representative units, one (represented in black dots) shows phase‐locked firing with respect to theta oscillations, whereas the other (represented in gray dots) does not. The peak coherence of the two units reached 43.93% and 6.55% at 8 Hz, respectively (Figure 2K). We counted the number of FS and RS units which showed a peak of spike‐LFP coherence higher than 20%, and found that the peak coherence value dominantly distributes in the theta band (Figure 2L,M,O,P). MK‐801 treatment did not alter the frequency distribution of peak spike‐LFP coherence, but reduced the coherence coefficients at theta band as shown by the cumulative distribution functions (CDFs, Figure 2N,Q).

### 3.3 Early postnatal MK‐801 treatment disrupts cellular calcium dynamics in individual hippocampal neurons

**FIGURE 3:**
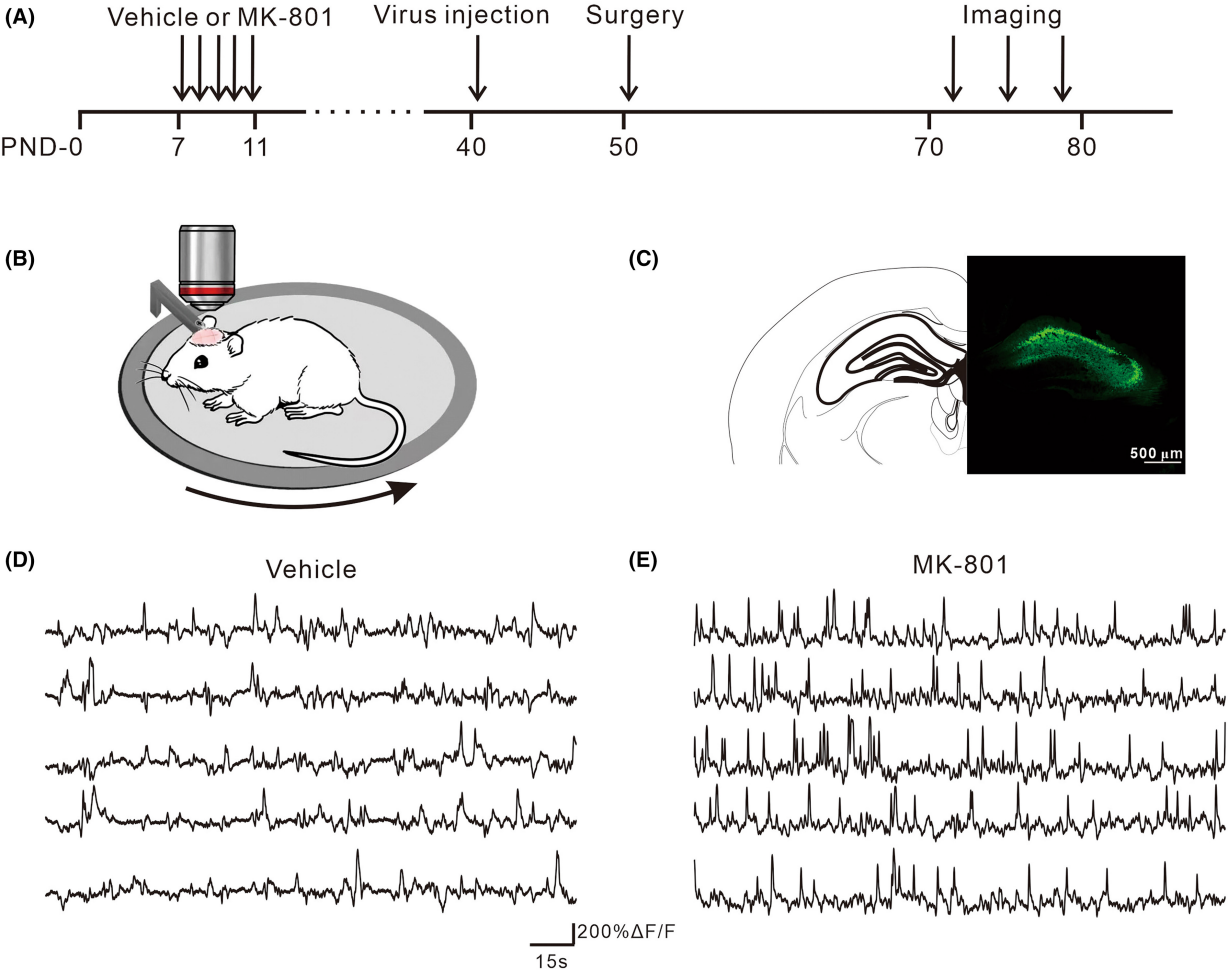
In vivo calcium imaging in dCA1 and calcium transients during locomotion. (A) The timeline of the experimental procedures for in vivo calcium imaging. (B) Mice head‐fixed and allowed to run freely on a treadmill. (C) Immunohistological image showing GCaMP6s expression in the dCA1. (D, E) Representative calcium traces of vehicle and MK‐801‐treated mice. Each line represents the calcium transient of one cell.

To image the neural activity, a virus encoding the calcium indicator for glutamatergic neurons (rAAV1‐CamKII‐GCaMP6s) was targeted unilaterally to the dorsal CA1 area in both MK‐801‐treated and vehicle mice on PND‐40. Chronic cranial window surgery was conducted at the site of hippocampal dCA1 on PND‐50, and calcium dynamics in individual hippocampal neurons were recorded on PND‐70 while monitoring the locomotion in the head‐fixed mice (Figure 3A–C). As showed by the example individual neurons (Figure 3D,E), the rate and amplitude of neural calcium transients were higher in MK‐801‐treated mice than the vehicle mice.

**FIGURE 4:**
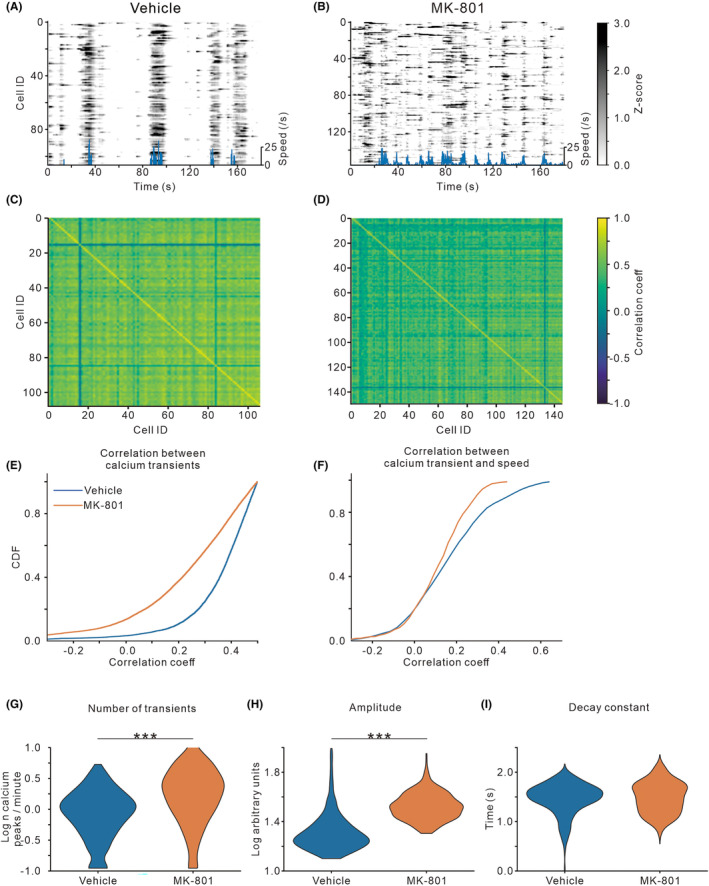
Effects of early postnatal MK‐801 treatment on calcium activities of individual hippocampal neurons. (A, B) Raster plot of *z*‐scored calcium activities imaged from the dCA1 neurons of one example mouse. Blue line below showing locomotion speed. (C, D) Heat map displaying representative correlation matrices of calcium activities between pairs of neurons in vehicle and MK‐801‐treated mice. (E, F) Line plot displaying cumulative distribution of pairwise correlation coefficients of calcium activities between individual neurons, and between the neural calcium activities and locomotion speed. (G–I) Violin plots quantifying the number, amplitude, and decay of detected calcium transients. Values are from four mice (two male/two female) per group. ***p < 0.001, Student's *t*‐test.

Figure 4A,B present the raster plots of *z*‐scored calcium transients of the example neurons. In the vehicle mice, a large proportion of neural calcium dynamics showed a high level of synchronization coupling to locomotion. However, the calcium dynamics had less synchronization across neurons in the MK‐801‐treated mice. First, we calculated Fisher‐corrected Pearson pairwise correlation of calcium dynamics between individual neurons (Figure 4C,D), and plotted the CDFs of correlation coefficients (Figure 4E). These results indicated that MK‐801 treatment reduced pairwise correlations between neural activities. We further analyzed the correlation between the neural calcium dynamics and locomotion speed, and found that MK‐801 treatment reduced the correlations coefficients between them (Figure 4F). Finally, we analyzed the rate (i.e., the number of transients, Figure 4G), amplitude (Figure 4H), and duration (i.e., the decay constant) (Figure 4I) of calcium transients averaged across all imaging sessions. Comparing to the vehicle group, MK‐801‐treated group showed higher rates (*t*_967_ = 10.10, *p* < 0.001) and amplitudes (*t*_967_ = 22.49, *p* < 0.001) but similar durations of calcium transients (*t*_967_ = 1.03, *p* = 0.30).

### 3.4 Therapeutic effects of ARI on hippocampal neural activities and hyperlocomotion in MK‐801‐treated mice

**FIGURE 5:**
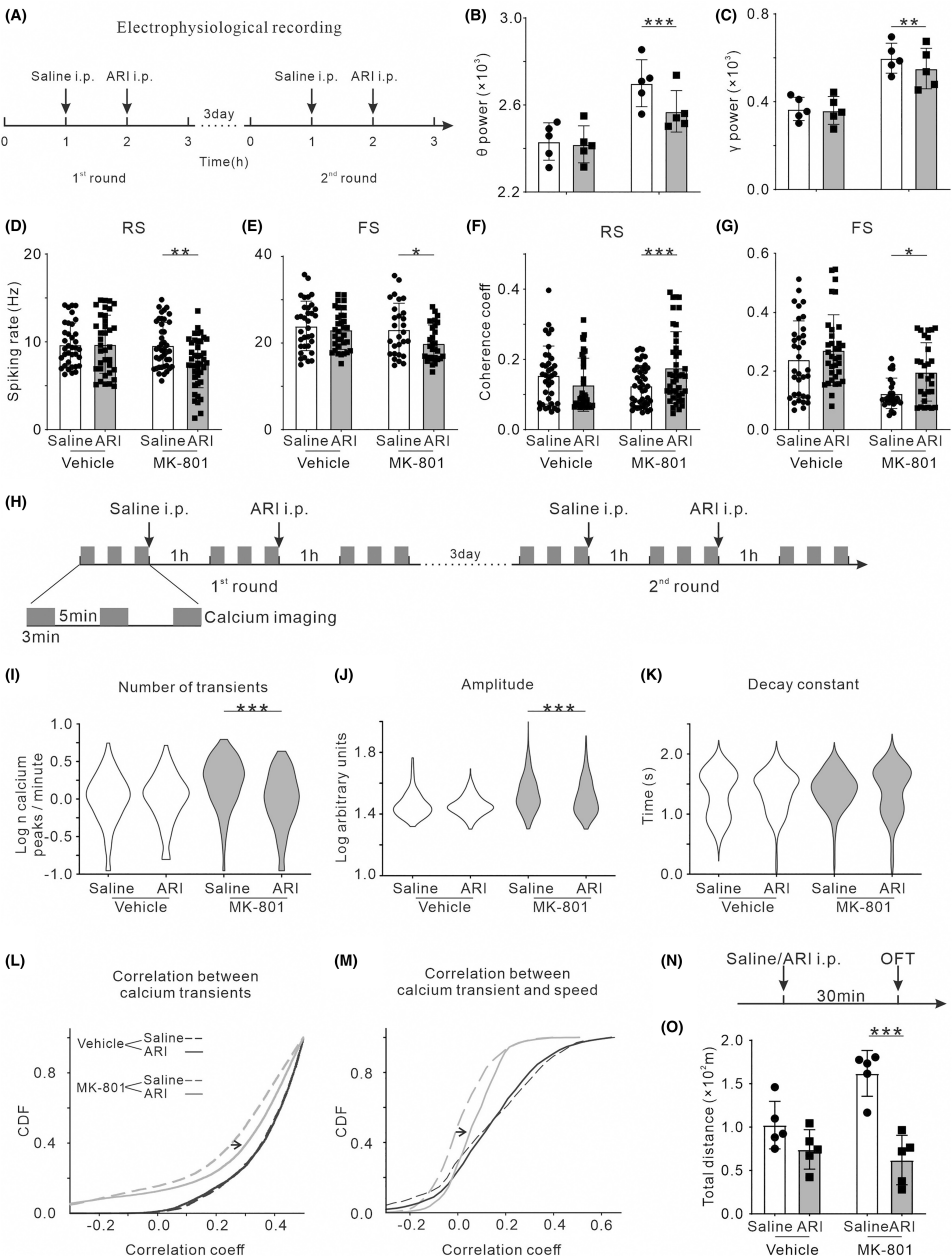
Effects of ARI treatment on the hippocampal neural activities and hyperlocomotion in MK‐801‐treated mice. For neural electrophysiological activities, (A) timeline of the experimental procedures of electrophysiological recording, (B) theta power of LFP (*n* = 5; three male/two female), (C) gamma power of LFP (*n* = 5; three male/two female), (D) spiking rate of RS unit (*n* = 36/42 from eight vehicle/MK‐801‐treated mice; four male/four female), (E) spiking rate of FS unit (*n* = 34/28 from eight vehicle/MK‐801‐treated mice; four male/four female), (F) coherence coefficients between spiking activity of RS unit and theta oscillation and (G) coherence coefficients between spiking activity of FS unit and theta oscillation. For calcium dynamics, (H) timeline of the experimental procedures of calcium imaging, (I) number, (J), amplitude, and (K) decay of detected calcium transients, (L) pairwise correlation of calcium activities between individual neurons and (M) correlation coefficients between the calcium activities and locomotion speed (*n* = 4; two male/two female). For locomotor activity, (N) timeline of the experimental procedures of OFT, (O) total traveled distance in OFT (*n* = 5; three male/two female). *p < 0.05, **p < 0.01, ***p < 0.001, Two‐way ANOVA with Šídák's post‐hoc test.

Furthermore, we evaluated the therapeutic effects of ARI on the electrophysiological activities of MK‐801‐treated mice (Figure 5A). Two‐way ANOVA with Šídák's post‐hoc test revealed that theta powers were significantly different between groups (*F*_1,8_ = 13.39, *p* = 0.006), treatment (*F*_1,8_ = 19.81, *p* = 0.002) and interaction (*F*_1,8_ = 13.27, *p* = 0.007), in the MK‐801 group, theta power was significantly reduced by ARI treatment (post‐hoc, *t*_8_ = 5.72, *p* < 0.001, Figure 5B). Similarly, gamma powers were significantly reduced by ARI treatment in the MK‐801 group (*F*_group (1,8)_ = 22.87, *p* = 0.001; *F*_treatment (1,8)_ = 13.56, *p* = 0.006; *F*_interation (1,8)_ = 7.68, *p* = 0.02; post‐hoc, *t*_8_ = 4.56, *p* = 0.004, Figure 5C). The spontaneous spike activity of RS and FS units were significantly reduced by ARI treatment in the MK‐801 group. (post‐hoc, *t*_152_ = 3.38, *p* = 0.006, Figure 5D; *t*_120_ = 3.05, *p* = 0.02, Figure 5E). We, then, examined the effect on coherence coefficients between spiking activity and theta oscillation. ARI injection increased the coherence coefficients between RS and theta oscillation in the MK‐801‐treated mice (post‐hoc, *t*_152_ = 4.02, *p* < 0.001, Figure 5F), as well as between FS and theta oscillation (post‐hoc, *t*_120_ = 2.89, *p* = 0.03, Figure 5G).

Next, we used calcium imaging to evaluate the effect of ARI on the activities of individual hippocampal neurons (Figure 5H). ARI injection reduced the rate in MK‐801‐treated mice (*F*_group (11362)_ = 25.21, *p* < 0.001; *F*_treatment (11362)_ = 39.12, *p* < 0.001; *F*_interation (11362)_ = 38.49, *p* < 0.001; post‐hoc, *t*_1362_ = 8.81, *p* < 0.001, Figure 5I), but not in the vehicle mice (*t*_1362_ = 0.04, *p* > 0.99). Amplitude of calcium transients was significantly decreased in MK‐801‐treated mice (post‐hoc, *t*_1362_ = 5.62, *p <* 0.001, Figure 5J), but not in the vehicle mice (*t*_1362_ = 0.01, *p* > 0.99). The duration of calcium transients was not affected by ARI (*F*_group (11362)_ = 3.73, *p* = 0.054; *F*_treatment (11362)_ = 0.14, *p* = 0.71; *F*_interation (11362)_ = 0.32, *p* = 0.57, Figure 5K). Comparing to saline injection, ARI injection had no effect on pairwise correlations of calcium dynamics between individual neurons in vehicle mice (black solid line and dash line in Figure 5L). And the correlations between the neural calcium dynamics and locomotion speed was not affected by ARI injection (black solid line and dash line in Figure 5M). In contrast, ARI injection in the MK‐801‐treated mice significantly increased pairwise correlations of calcium dynamics between individual neurons (gray solid line and dash line in Figure 5L) and the those between the neural calcium dynamics and locomotion speed (gray solid line and dash line in Figure 5M).

The OFT was conducted to elucidate the role of ARI on the locomotion impairment associated with MK‐801 treatment. Both the vehicle and MK‐801‐treated mice were randomly divided into two groups (five in each): administered with saline and ARI. The OFT was conducted at 30 min after the drug administration (Figure 5N). In MK‐801‐treated mice, the locomotor hyperactivity in the OFT was significantly reduced by the injection of ARI (post‐hoc, *t*_16_ = 5.99, *p* < 0.001, Figure 5O), whereas no significant changes were observed in vehicle mice (*t*_16_ = 1.68, *p* = 0.51).

## 4 DISCUSSION

The goal of the present study was to test whether early postnatal MK‐801 treatment, a well‐established model of SZ, alters the locomotor activity, neural electrophysiological activities and calcium dynamics, and evaluate the potential therapeutic effects of ARI. Our results confirmed that early postanal MK‐801 treatment could drive hyperlocomotion, and increase the theta power of LFP in hippocampus. Furthermore, we found that early postanal MK‐801 treatment reduced the correlation coefficient between the hippocampal spiking activity and theta oscillation. Consistently, the calcium dynamics of hippocampal neurons desynchronized and lost the correlation to locomotion speed, even though the rate and amplitude of calcium transients were increased. ARI corrected all the measured abnormalities of neural electrophysiological activities and calcium dynamics. Therefore, this study reconciles experimental approaches in neuroscience to comprehensively assess the neurophysiological changes of SZ model, and evaluate the therapeutic effect of ARI.

It has been well established that glutamatergic neurotransmission and more particularly that mediated by NMDAR, is disturbed in SZ.^25^, ^26^ Therefore, NMDAR antagonists, such as MK‐801, have been largely employed as experimental models of psychosis in rodents.^27^, ^28^, ^29^ Consistent with previous reports, our current results show that postnatal injection of MK‐801 in mice could cause hyperlocomotion.^30^, ^31^ We further found that the power of hippocampal theta oscillation was increased in MK‐801‐treated mice. Our study for the first time observed that spike activities of hippocampal neurons were less locked to the phase of theta oscillation in MK‐801‐treated mice. This decreased phase locking was not accompanied by any alterations in the frequency range of theta oscillation. That is, neurons maintained a reduced phase relationship with the theta oscillations, which may reflect a disruption of firing coordination within neuron clusters. Gamma powers of LFP in hippocampus were also increased in MK‐801‐treated mice, which is consistent with previous studies in NMDAR hypofunction models.^14^, ^32^, ^33^ Gamma activities in the hippocampus have been reported to correlate with arousal and active waking.^34^, ^35^ Under normal conditions, hippocampal gamma activity was higher during active behaviors, such as walking, as compared to awake immobility.^35^, ^36^ Additionally, our study employed calcium imaging to investigate the activities of hippocampal CA1 neural network in the MK‐801‐treated mice, and found that the amplitude and rate of calcium transients were increased but the synchrony was decreased, the correlation between calcium transient and locomotion speed was reduced.

Theta oscillation is a prominent pattern of hippocampal neural electrical activity, which is generated by the medial septum (MS) and can recruit hippocampal neurons to discharge synchronously at theta rhythm.^37^, ^38^, ^39^ Because theta oscillation closely correlates with locomotion, there is also a correlation between hippocampal neuron activity and locomotion.^40^, ^41^, ^42^, ^43^, ^44^ The coupling between neural activities and theta oscillation plays an important role in spatial navigation by facilitating the processing of spatial information during movement.^24^, ^45^, ^46^ Previous studies have shown that MK‐801 treatment can disrupt synaptic transmission between hippocampal neurons,^17^ thereby reducing the ability of neurons to coordinate with each other and decreasing the coherence to theta oscillation. Consequently, the mutual correlations between neural activities, theta oscillation, and locomotion are disrupted, and the hippocampal function to encode spatial information during movement is impaired.

Moreover, we evaluated the therapeutic effects of ARI on neural electrophysiological activities, calcium dynamics and behaviors. Our results indicated that ARI could correct all the measured abnormalities of neural electrophysiological activities, calcium dynamics and the locomotor deficits of MK‐801‐treated mice. ARI is an atypical antipsychotic that acts as an antagonist of both 5‐HT2A and postsynaptic dopamine type 2 (D2) receptors as well as a partial agonist of 5‐HT1A and presynaptic D2 receptors.^47^, ^48^ And ARI displays a higher affinity for the dopamine receptor than the serotonin receptor.^49^, ^50^ Hippocampal hyperactivity has been reported to increase dopamine release from the ventral tegmental area (VTA) to the NAc,^51^ which is associated with hyperlocomotion.^52^ This regulation may occur through a disinhibition of dopamine neuron activity in the VTA. It has been proposed that hippocampus modulates dopamine neuron population activity via a polysynaptic projection involving a glutamatergic input to the NAc that increases GABAergic activity to the ventral pallidum (VP). This increase in GABA activity decreases tonic VP activity, resulting in a disinhibition of dopamine neuron activity in the VTA.^53^, ^54^ Conversely, the hyperfunction of dopamine system underlies hyperresponsive and dysrhythmic activities in the hippocampus of SZ patients and animal models.^55^ Consistent with previous studies,^56^, ^57^ we confirmed that ARI could reduce the locomotor hyperactivity in our SZ model. This may be due to the stabilizing effect of ARI on the dopaminergic activities of NAc and VTA. Consequently, their interference with hippocampal neuron activity was eliminated.

## 5 CONCLUSION

The hyperactivity and dysrhythmia of the hippocampal neurons in the model mice may correlate with the hyperlocomotion. And ARI ameliorated perturbations produced by the postnatal MK‐801 treatment.

## CONFLICT OF INTEREST STATEMENT

The authors declare no competing financial interests.

## Supporting information


Data S1

## Data Availability

The data used to support the findings of this study are available from the corresponding author upon reasonable request.

## References

[1] Sarkar S , Grover S . Antipsychotics in children and adolescents with schizophrenia: a systematic review and meta‐analysis. Indian J Pharmacol. 2013;45(5):439‐446. 24130376 10.4103/0253-7613.117720 PMC3793512

[2] Rajji TK , Miranda D , Mulsant BH . Cognition, function, and disability in patients with schizophrenia: a review of longitudinal studies. Can J Psychiatr. 2014;59(1):13‐17. 10.1177/070674371405900104 PMC4079219 24444319

[3] Kempton MJ , Stahl D , Williams SC , DeLisi LE . Progressive lateral ventricular enlargement in schizophrenia: a meta‐analysis of longitudinal MRI studies. Schizophr Res. 2010;120(1–3):54‐62. 20537866 10.1016/j.schres.2010.03.036

[4] Schultz SK , Andreasen NC . Schizophrenia. Lancet. 1999;353(9162):1425‐1430. 10227239 10.1016/s0140-6736(98)07549-7

[5] Abi‐Dargham A . Do we still believe in the dopamine hypothesis? New data bring new evidence. Int J Neuropsychopharmacol. 2004;7(Suppl 1):S1‐S5. 10.1017/S1461145704004110 14972078

[6] Laruelle M , Abi‐Dargham A . Dopamine as the wind of the psychotic fire: new evidence from brain imaging studies. J Psychopharmacol. 1999;13(4):358‐371. 10667612 10.1177/026988119901300405

[7] Blaha CD , Yang CR , Floresco SB , Barr AM , Phillips AG . Stimulation of the ventral subiculum of the hippocampus evokes glutamate receptor‐mediated changes in dopamine efflux in the rat nucleus accumbens. Eur J Neurosci. 1997;9(5):902‐911. 9182943 10.1111/j.1460-9568.1997.tb01441.x

[8] Legault M , Wise RA . Injections of *N*‐methyl‐d‐aspartate into the ventral hippocampus increase extracellular dopamine in the ventral tegmental area and nucleus accumbens. Synapse. 1999;31(4):241‐249. 10051104 10.1002/(SICI)1098-2396(19990315)31:4<241::AID-SYN1>3.0.CO;2-#

[9] Verma S , Sitoh YY , Ho YC , et al. Hippocampal volumes in first‐episode psychosis. J Neuropsychiatry Clin Neurosci. 2009;21(1):24‐29. 19359448 10.1176/jnp.2009.21.1.24

[10] Talbot K , Eidem WL , Tinsley CL , et al. Dysbindin‐1 is reduced in intrinsic, glutamatergic terminals of the hippocampal formation in schizophrenia. J Clin Invest. 2004;113(9):1353‐1363. 15124027 10.1172/JCI20425 PMC398430

[11] Kätzel D , Wolff AR , Bygrave AM , Bannerman DM . Hippocampal hyperactivity as a druggable circuit‐level origin of aberrant salience in schizophrenia. Front Pharmacol. 2020;11:486811. 33178010 10.3389/fphar.2020.486811 PMC7596262

[12] Gallinat J , McMahon K , Kühn S , Schubert F , Schaefer M . Cross‐sectional study of glutamate in the anterior cingulate and hippocampus in schizophrenia. Schizophr Bull. 2016;42(2):425‐433. 26333842 10.1093/schbul/sbv124 PMC4753596

[13] Kawabe K , Miyamoto E . Effects of early postnatal MK‐801 treatment on behavioral properties in rats: differences according to treatment schedule. Behav Brain Res. 2019;370:111926. 31029708 10.1016/j.bbr.2019.111926

[14] Plataki ME , Diskos K , Sougklakos C , et al. Effect of neonatal treatment with the NMDA receptor antagonist, MK‐801, during different temporal windows of postnatal period in adult prefrontal cortical and hippocampal function. Front Behav Neurosci. 2021;15:689193. 34177484 10.3389/fnbeh.2021.689193 PMC8230549

[15] Uehara T , Sumiyoshi T , Seo T , et al. Neonatal exposure to MK‐801, an *N*‐methyl‐d‐aspartate receptor antagonist, enhances methamphetamine‐induced locomotion and disrupts sensorimotor gating in pre‐ and postpubertal rats. Brain Res. 2010;1352:223‐230. 20633540 10.1016/j.brainres.2010.07.013

[16] Howe AG , Blair HT . Modulation of lateral septal and dorsomedial striatal neurons by hippocampal sharp‐wave ripples, theta rhythm, and running speed. Hippocampus. 2022;32(3):153‐178. 34918836 10.1002/hipo.23398 PMC9299855

[17] Hernández‐Frausto M , López‐Rubalcava C , Galván EJ . Progressive alterations in synaptic transmission and plasticity of area CA1 precede the cognitive impairment associated with neonatal administration of MK‐801. Neuroscience. 2019;404:205‐217. 30703507 10.1016/j.neuroscience.2019.01.036

[18] Abad‐Perez P , Molina‐Payá FJ , Martínez‐Otero L , Borrell V , Redondo RL , Brotons‐Mas JR . Theta/gamma Co‐modulation disruption after NMDAr blockade by MK‐801 is associated with spatial working memory deficits in mice. Neuroscience. 2023;519:162‐176. 36990270 10.1016/j.neuroscience.2023.03.022 PMC10229075

[19] Shapiro DA , Renock S , Arrington E , et al. Aripiprazole, a novel atypical antipsychotic drug with a unique and robust pharmacology. Neuropsychopharmacology. 2003;28(8):1400‐1411. 12784105 10.1038/sj.npp.1300203

[20] Ribeiro ELA , de Mendonça Lima T , Vieira MEB , Storpirtis S , Aguiar PM . Efficacy and safety of aripiprazole for the treatment of schizophrenia: an overview of systematic reviews. Eur J Clin Pharmacol. 2018;74(10):1215‐1233. 29905899 10.1007/s00228-018-2498-1

[21] Leite JV , Guimarães FS , Moreira FA . Aripiprazole, an atypical antipsychotic, prevents the motor hyperactivity induced by psychotomimetics and psychostimulants in mice. Eur J Pharmacol. 2008;578(2–3):222‐227. 18021764 10.1016/j.ejphar.2007.09.016

[22] Bokil H , Andrews P , Kulkarni JE , Mehta S , Mitra PP . Chronux: a platform for analyzing neural signals. J Neurosci Methods. 2010;192(1):146‐151. 20637804 10.1016/j.jneumeth.2010.06.020 PMC2934871

[23] Yang W , Chini M , Popplau JA , et al. Anesthetics fragment hippocampal network activity, alter spine dynamics, and affect memory consolidation. PLoS Biol. 2021;19(4):e3001146. 33793545 10.1371/journal.pbio.3001146 PMC8016109

[24] Pettit NL , Yap EL , Greenberg ME , Harvey CD . Fos ensembles encode and shape stable spatial maps in the hippocampus. Nature. 2022;609(7926):327‐334. 36002569 10.1038/s41586-022-05113-1 PMC9452297

[25] Bubeníková‐Valesová V , Horácek J , Vrajová M , Höschl C . Models of schizophrenia in humans and animals based on inhibition of NMDA receptors. Neurosci Biobehav Rev. 2008;32(5):1014‐1023. 18471877 10.1016/j.neubiorev.2008.03.012

[26] Javitt DC , Zukin SR . Recent advances in the phencyclidine model of schizophrenia. Am J Psychiatry. 1991;148(10):1301‐1308. 1654746 10.1176/ajp.148.10.1301

[27] Kruk‐Slomka M , Biala G . Cannabidiol attenuates MK‐801‐induced cognitive symptoms of schizophrenia in the passive avoidance test in mice. Molecules. 2021;26(19):5977. 34641522 10.3390/molecules26195977 PMC8513030

[28] Sun ZY , Gu LH , Ma DL , et al. Behavioral and neurobiological changes in a novel mouse model of schizophrenia induced by the combination of cuprizone and MK‐801. Brain Res Bull. 2021;174:141‐152. 34119597 10.1016/j.brainresbull.2021.06.007

[29] Akosman MS , Türkmen R , Demirel HH . Investigation of the protective effect of resveratrol in an MK‐801‐induced mouse model of schizophrenia. Environ Sci Pollut Res Int. 2021;28(46):65872‐65884. 34322799 10.1007/s11356-021-15664-x

[30] Saeedi Goraghani M , Ahmadi‐Zeidabadi M , Bakhshaei S , et al. Behavioral consequences of simultaneous postnatal exposure to MK‐801 and static magnetic field in male Wistar rats. Neurosci Lett. 2019;701:77‐83. 30790646 10.1016/j.neulet.2019.02.026

[31] Nozari M , Shabani M , Hadadi M , Atapour N . Enriched environment prevents cognitive and motor deficits associated with postnatal MK‐801 treatment. Psychopharmacology. 2014;231(22):4361‐4370. 24770628 10.1007/s00213-014-3580-8

[32] Lemercier CE , Holman C , Gerevich Z . Aberrant alpha and gamma oscillations ex vivo after single application of the NMDA receptor antagonist MK‐801. Schizophr Res. 2017;188:118‐124. 28109667 10.1016/j.schres.2017.01.017

[33] Kehrer C , Dugladze T , Maziashvili N , et al. Increased inhibitory input to CA1 pyramidal cells alters hippocampal gamma frequency oscillations in the MK‐801 model of acute psychosis. Neurobiol Dis. 2007;25(3):545‐552. 17169567 10.1016/j.nbd.2006.10.015

[34] Jones BE . Activity, modulation and role of basal forebrain cholinergic neurons innervating the cerebral cortex. Prog Brain Res. 2004;145:157‐169. 14650914 10.1016/S0079-6123(03)45011-5

[35] Leung LS . Generation of theta and gamma rhythms in the hippocampus. Neurosci Biobehav Rev. 1998;22(2):275‐290. 9579318 10.1016/s0149-7634(97)00014-6

[36] Leung LS . Nonlinear feedback model of neuronal populations in hippocampal CAl region. J Neurophysiol. 1982;47(5):845‐868. 7086472 10.1152/jn.1982.47.5.845

[37] Dannenberg H , Pabst M , Braganza O , et al. Synergy of direct and indirect cholinergic septo‐hippocampal pathways coordinates firing in hippocampal networks. J Neurosci. 2015;35(22):8394‐8410. 26041909 10.1523/JNEUROSCI.4460-14.2015 PMC6605336

[38] Lawson VH , Bland BH . The role of the septohippocampal pathway in the regulation of hippocampal field activity and behavior: analysis by the intraseptal microinfusion of carbachol, atropine, and procaine. Exp Neurol. 1993;120(1):132‐144. 8477826 10.1006/exnr.1993.1047

[39] Newman EL , Gillet SN , Climer JR , Hasselmo ME . Cholinergic blockade reduces theta‐gamma phase amplitude coupling and speed modulation of theta frequency consistent with behavioral effects on encoding. J Neurosci. 2013;33(50):19635‐19646. 24336727 10.1523/JNEUROSCI.2586-13.2013 PMC3858632

[40] Belchior H , Lopes‐Dos‐Santos V , Tort AB , Ribeiro S . Increase in hippocampal theta oscillations during spatial decision making. Hippocampus. 2014;24(6):693‐702. 24520011 10.1002/hipo.22260 PMC4229028

[41] Burgess N , O'Keefe J . Models of place and grid cell firing and theta rhythmicity. Curr Opin Neurobiol. 2011;21(5):734‐744. 21820895 10.1016/j.conb.2011.07.002 PMC3223517

[42] Hernández‐Pérez JJ , Gutiérrez‐Guzmán BE , Olvera‐Cortés ME . Hippocampal strata theta oscillations change their frequency and coupling during spatial learning. Neuroscience. 2016;337:224‐241. 27615031 10.1016/j.neuroscience.2016.09.003

[43] Korotkova T , Ponomarenko A , Monaghan CK , et al. Reconciling the different faces of hippocampal theta: the role of theta oscillations in cognitive, emotional and innate behaviors. Neurosci Biobehav Rev. 2018;85:65‐80. 28887226 10.1016/j.neubiorev.2017.09.004

[44] Bender F , Gorbati M , Cadavieco MC , et al. Theta oscillations regulate the speed of locomotion via a hippocampus to lateral septum pathway. Nat Commun. 2015;6:8521. 26455912 10.1038/ncomms9521 PMC4633825

[45] Rolotti SV , Blockus H , Sparks FT , Priestley JB , Losonczy A . Reorganization of CA1 dendritic dynamics by hippocampal sharp‐wave ripples during learning. Neuron. 2022;110(6):977‐991 e974. 35041805 10.1016/j.neuron.2021.12.017 PMC8930454

[46] Cholvin T , Hainmueller T , Bartos M . The hippocampus converts dynamic entorhinal inputs into stable spatial maps. Neuron. 2021;109(19):3135‐3148.e3137. 34619088 10.1016/j.neuron.2021.09.019 PMC8516433

[47] Casey AB , Canal CE . Classics in chemical neuroscience: aripiprazole. ACS Chem Neurosci. 2017;8(6):1135‐1146. 28368577 10.1021/acschemneuro.7b00087 PMC5495458

[48] Allen JA , Yost JM , Setola V , et al. Discovery of β‐arrestin‐biased dopamine D2 ligands for probing signal transduction pathways essential for antipsychotic efficacy. Proc Natl Acad Sci USA. 2011;108(45):18488‐18493. 22025698 10.1073/pnas.1104807108 PMC3215024

[49] Stępnicki P , Kondej M , Kaczor AA . Current concepts and treatments of schizophrenia. Molecules. 2018;23(8):2087. 30127324 10.3390/molecules23082087 PMC6222385

[50] Maini K , Gould H , Hicks J , et al. Aripiprazole Lauroxil, a novel injectable long‐acting antipsychotic treatment for adults with schizophrenia: a comprehensive review. Neurol Int. 2021;13(3):279‐296. 34287335 10.3390/neurolint13030029 PMC8293312

[51] Floresco SB , Todd CL , Grace AA . Glutamatergic afferents from the hippocampus to the nucleus accumbens regulate activity of ventral tegmental area dopamine neurons. J Neurosci. 2001;21(13):4915‐4922. 11425919 10.1523/JNEUROSCI.21-13-04915.2001 PMC6762358

[52] White IM , Whitaker C , White W . Amphetamine‐induced hyperlocomotion in rats: hippocampal modulation of the nucleus accumbens. Hippocampus. 2006;16(7):596‐603. 16763995 10.1002/hipo.20189

[53] Floresco SB , West AR , Ash B , Moore H , Grace AA . Afferent modulation of dopamine neuron firing differentially regulates tonic and phasic dopamine transmission. Nat Neurosci. 2003;6(9):968‐973. 12897785 10.1038/nn1103

[54] Lodge DJ , Grace AA . Hippocampal dysregulation of dopamine system function and the pathophysiology of schizophrenia. Trends Pharmacol Sci. 2011;32(9):507‐513. 21700346 10.1016/j.tips.2011.05.001 PMC3159688

[55] Grace AA , Gomes FV . The circuitry of dopamine system regulation and its disruption in schizophrenia: insights into treatment and prevention. Schizophr Bull. 2019;45(1):148‐157. 29385549 10.1093/schbul/sbx199 PMC6293217

[56] Hereta M , Kaminska K , Rogoz Z . Co‐treatment with antidepressants and aripiprazole reversed the MK‐801‐induced some negative symptoms of schizophrenia in rats. Pharmacol Rep. 2019;71(5):768‐773. 31351318 10.1016/j.pharep.2019.04.007

[57] Rogoz Z , Wasik A , Lorenc‐Koci E . Combined treatment with aripiprazole and antidepressants reversed some MK‐801‐induced schizophrenia‐like symptoms in mice. Pharmacol Rep. 2018;70(4):623‐630. 29885435 10.1016/j.pharep.2018.02.022

