# Supplementary material for: Correlation between desynchrony of hippocampal neural activity and hyperlocomotion in the model mice of schizophrenia and therapeutic effects of aripiprazole
Source: CNS Neurosci Ther. 2024 May 3;30(5):e14739. doi: 10.1111/cns.14739 (PMC11069053; doi:10.1111/cns.14739)
Supplement: Supplementary file 1 — Data S1 [file CNS-30-e14739-s001.docx]

**Materials and methods**

*Experiment Procedures*

To evaluate the motor function of mice, OFT was conducted. One hour following OFT, the mice were sacrificed and the brain sections were collected for analysis of hippocampal neuronal activity during locomotion with c-Fos immunolabeling.

For recording local field potentials and spike activity, the MK-801 treated and vehicle mice underwent electrode implantation in field CA1 of the hippocampus on PND-60. After recovering, electrophysiological recordings were conducted from PND-70 to PND-80. For each mouse, we firstly recorded the spike activities and LFPs in the hippocampus for 1 h baseline period, then intraperitoneally injected saline and recorded the second 1 h period, lastly injected ARI (MedChemExpress, Monmouth Junction, NJ, USA) at a dose of 1 mg/kg (i.p.), and recorded the third 1 h period. After 3 days for washing out, drug injection and electrophysiological recording experiments were repeated. Each mouse received 2 rounds of ARI injection. After recordings, the recording sites were confirmed by histological tissue processing. Only the data recorded from the dCA1 region were analyzed, those outside the dCA1 were excluded. Because there were no obvious differences between the results of the first and second recordings, we pooled them together for further analyses.

To image neural activity, a virus encoding the calcium indicator for glutamatergic neurons (rAAV1-CamKII-GCaMP6s, BrainVTA, Wuhan, China) was targeted unilaterally to the dorsal CA1 area in both MK-801-treated and vehicle mice on PND-40. Chronic cranial window surgery was conducted at the site of hippocampal dCA1 on PND-50, and calcium dynamics in individual hippocampal neurons were recorded from PND-70 while monitoring the locomotion in the head-fixed mice. Each day mice were firstly subjected to a baseline imaging experiment (3×3 min imaging with 5min interval), then received an injection of saline followed by an imaging experiment at 1h later, lastly received injection of ARI followed by an imaging experiment at 1h later. After 3 days for washing out, drug injection and imaging experiments were repeated. Each mouse received 2 rounds of ARI injection. Because there were no obvious differences between the results of the first and second recordings, we pooled them together for further analyses.

*Surgical procedures for in vivo electrophysiological recording*

Surgery was conducted at least 1 week before recordings. Mice were anesthetized with isoflurane (3%–4% isoflurane for induction, 1%–2% isoflurane for maintenance). Body temperature was maintained at approximately 37˚C using a heating blanket. Eyes were covered with eye ointment to prevent drying. Subsequently, mice were fixed in a stereotaxic apparatus (#68001, RWD Life science, Shenzhen, China), the fur was removed with a fine trimmer, and the skin of the head was disinfected with medical alcohol. The skin of the head was removed and the skull was cleaned using a bone scraper. Two stainless screws were separately inserted into the cranium above cerebellum. The silver wires soldered to a pin connector were connected with stainless screws and served as ground and reference. Two metal headposts were attached to the skull with dental cement. The locations of bilateral hippocampus (AP = - 2.0 mm, ML = ± 2.0 mm) were marked on the surface of skull by ink. After recovery from anesthesia, mice were returned to their home cage, and antibiotics and analgesic medicines were added to their drinking water.

*Virus injection and hippocampal window surgery for in vivo calcium imaging*

Mice were anesthetized and fixed in a stereotaxic apparatus with the procedures similar above. The skin was removed by a midline scalp incision and a small hole (~2mm diameter) was drilled over the hippocampus CA1 with a dental drill. A genetically encoded calcium indicator, rAAV-CaMKIIα-GCaMP6s-WPRE-hGH (BrainVTA, Wuhan, China) for glutamatergic neural population, was targeted unilaterally to the CA1 area (∼0.3 μL/injection, ∼10 minutes/injection) at a depth of 1.5mm below the surface of the skull using a beveled glass micropipette. After the injection, the pipette stayed in place for at least 5 minutes before it was withdrawn, and the skin was sutured. After recovery from anesthesia, mice were returned to their home cage, and antibiotics and analgesic medicines were added to their drinking water.

Around 10 days later, mice underwent another surgery to implant a hippocampal window. A dental drill was used to cut a ∼4mm diameter craniotomy centered over the previously made small hole over hippocampus and the dura was removed with forceps. Then, the somatosensory cortex above the hippocampus were carefully aspirated until the white matter tracts of the corpus callosum became visible. The craniotomy was washed with sterile PBS, and a metal cannula with a coverslip sealing one of the openings was inserted over the dCA1. Two stainless screws were separately inserted into cranium above cerebellum, and the stainless steel headposts were attached to the skull with dental cement. After recovery from anesthesia, mice were returned to their home cage, and antibiotics and analgesic medicines were added to their drinking water. The mice were allowed to recover from the surgery for at least 20 days before the imaging sessions began.

*Immunofluorescence*

We checked the GCaMP6s expression of the mice to ensure the expression was restricted to dCA1. Mice were anesthetized with pentobarbital (100 mg/kg) and perfused with ~10 ml phosphate-buffered saline (PBS) followed by ~20 ml 4% paraformaldehyde in PBS. The brains were removed and immersed in 30% sucrose solution overnight before being sectioned at 20 μm thickness via a freezing microtome (CM1950; Leica, Wetzlar, Germany). The brain slices were then collected on glass slides.

To analyze neuronal activity in the hippocampus during locomotion, c-Fos immunolabeling was performed. PND-70 mice were sacrificed 1h after OFT and the brain sections were collected as described above. After being washed using PBS, sections were permeabilized for 30 min using 0.3% Triton X-100 at 37 °C. Sections were then blocked for 2 h using 10% goat serum, after which they were probed for 48 h with primary rabbit polyclonal anti-c-Fos (1:5000; Abcam Inc., Cambridge, MA, USA) at 4 °C. Sections were then rinsed thrice in PBS and were then placed in biotinylated goat anti-rabbit antibody (1:200; Proteintech, Wuhan, China) for two hours at room temperature. After three additional washes, sections were mounted and covered by a coverslip treated using an anti-fluorescence quenching medium. Tissue sections were examined using an Olympus microscope (CX41) equipped with a digital camera. The whole-brain slices were imaged under a microscope (BX53, Olympus, Tokyo, Japan).
